# Buffering Effects of Internet Use on Caregiving-Related Health Impacts and Loneliness Among Older Informal Caregivers in California: Cross-Sectional Study

**DOI:** 10.2196/74209

**Published:** 2025-11-27

**Authors:** Xiang Qi, Ruotong Liu, Eunjung Ko, Yaolin Pei, Bei Wu

**Affiliations:** 1 Rory Meyers College of Nursing, New York University, 433 1st Ave, New York, NY, 10010, United States, 1 6468988694, 1 6468988694; 2 School of Nursing, The University of Texas at Austin, Austin, TX, United States

**Keywords:** social isolation, social disconnection, family caregiving, information and communication technology, digital technology, stress-process model

## Abstract

**Background:**

Loneliness has emerged as a global public health issue, with recent data indicating that 27.6% of adults aged 65 to 80 report feelings of loneliness despite the postpandemic resumption of social activities. Older caregivers face unique challenges that may exacerbate feelings of loneliness due to the demanding nature of caregiving responsibilities. While internet use has been suggested as a potential intervention to reduce loneliness, its moderating effect on the relationship between caregiving-related health effects and loneliness remains understudied.

**Objective:**

This study aims to investigate: (1) the association between caregiving-related health effects and loneliness among older informal caregivers; (2) the relationship between internet use frequency and loneliness; and (3) whether internet use moderates the association between caregiving-related health effects and loneliness.

**Methods:**

We analyzed cross-sectional data from the 2019‐2020 California Health Interview Survey, focusing on 3957 informal caregivers aged 65 and older. Loneliness was measured using a modified 3-item UCLA Loneliness Scale. Health effects of caregiving were assessed by self-reported physical or mental health problems due to caregiving responsibilities. Internet use frequency was measured on a 4-point scale. Multivariable linear regressions were used to test the study aims, adjusting for sociodemographic factors, health status, and caregiving-context characteristics.

**Results:**

Among participants, 475 (12.0%) reported experiencing physical or mental health problems due to caregiving responsibilities. After adjusting for covariates, caregivers who experienced health problems related to caregiving reported higher levels of loneliness compared to those who did not (*β*=0.76, SE .07, *P*<.001). More frequent internet use was associated with a lower level of loneliness (*β*=−0.11, SE 0.03, *P*<.001). Additionally, internet use significantly moderated the relationship between caregiving-related health effects and loneliness (*β*=−.16, SE 0.07, *P*=.02), suggesting that the negative impact of caregiving-related health effects on loneliness was attenuated among caregivers who used the internet more frequently.

**Conclusions:**

Caregiving-related health effects are associated with increased loneliness among older informal caregivers, but more frequent internet use may both directly reduce loneliness and buffer against the adverse impact of caregiving on loneliness. These findings align with recent research highlighting the potential of technology-based interventions to combat social disconnection among older adults. Health care providers and policy makers should consider implementing programs that enhance internet access among older caregivers as part of comprehensive strategies to address loneliness in this vulnerable population.

## Introduction

Loneliness in older adults has emerged as a growing global public health concern, with recent data indicating that 27.6% of adults aged 65 to 80 continue to experience feelings of loneliness despite the postpandemic resumption of social activities [1]. Loneliness is associated with multiple adverse health outcomes, including depression, heart disease, cognitive decline, sleep disturbance, and premature mortality [2-5]. The US Surgeon General’s 2023 Advisory on social isolation and loneliness equated the health impact of lacking social connection to smoking 15 cigarettes daily, underscoring the severity of this issue [6].

The significance of this issue is magnified for older informal caregivers, who face unique challenges and heightened risks of loneliness due to the demands and stress associated with caregiving responsibilities [7,8]. A recent analysis revealed that 15% of older caregivers experienced loneliness, with caregivers of persons with dementia having 1.62 times higher odds of experiencing loneliness compared to other caregivers [9]. For older informal caregivers, their advanced age can intensify their vulnerability to adverse health effects stemming from the burden of caregiving. In addition, older informal caregivers, who provide unpaid care to their family members or friends, often experience isolation due to the restrictive nature of caregiving duties that limit social interactions [7,8]. This isolation can exacerbate those adverse health effects associated with caregiving, creating a cycle of declining mental and physical health [10]. Given these challenges, identifying accessible interventions to alleviate loneliness in older caregivers is crucial. Increasingly, internet use has been considered a potential means to maintain social connections for those with limited in-person interaction opportunities.

While research on the general population has shown mixed or even negative associations between the widespread adoption of the internet and digital communication technologies and loneliness [11-13], the unique circumstances of informal caregivers may create a different context for digital engagement [14,15]. Caregivers face specific mobility constraints and social limitations that may make internet-based connections particularly valuable. For older informal caregivers, the internet may offer dual benefits: serving as a source of caregiving information and emotional support while facilitating social connections that reduce feelings of loneliness [16]. Recent evidence suggests that internet use may buffer against the negative health impacts of loneliness for older informal caregivers [17], enhance life satisfaction through improved interpersonal connections [18], and provide access to specialized support communities [15]. A recent scoping review indicated that technology-based interventions have shown preliminary evidence for reducing social isolation and loneliness among informal caregivers of persons with dementia [15]. However, despite the potential of internet use to serve as a resource for this population, research examining the intersection of caregiving, internet use, and loneliness within this population remains limited. This study aims to fill these gaps by examining the associations between caregiving-related health effects, internet use, and feelings of loneliness among older informal caregivers in California. Furthermore, it investigates whether internet use moderates the relationship between caregiving-related health effects and loneliness.

Using the Stress Process Model as a theoretical framework [19], this study considers how internet use may function as a coping resource that buffers against the adverse impact of caregiving stressors on feelings of loneliness. The Stress Process Model posits that caregiving experiences involve primary stressors (direct pressures of caregiving) and secondary stressors (indirect pressures such as financial strain), which can contribute to negative health outcomes, including loneliness [19]. In this study, we interpret caregiving-related health problems as a secondary stressor within the Stress Process Model framework, representing the negative consequences of primary caregiving stressors on caregivers’ well-being. Within this framework, internet use may serve as a coping resource that provides both informational and emotional support, potentially mitigating the impact of caregiving-related stress on loneliness.

**Figure 1.**
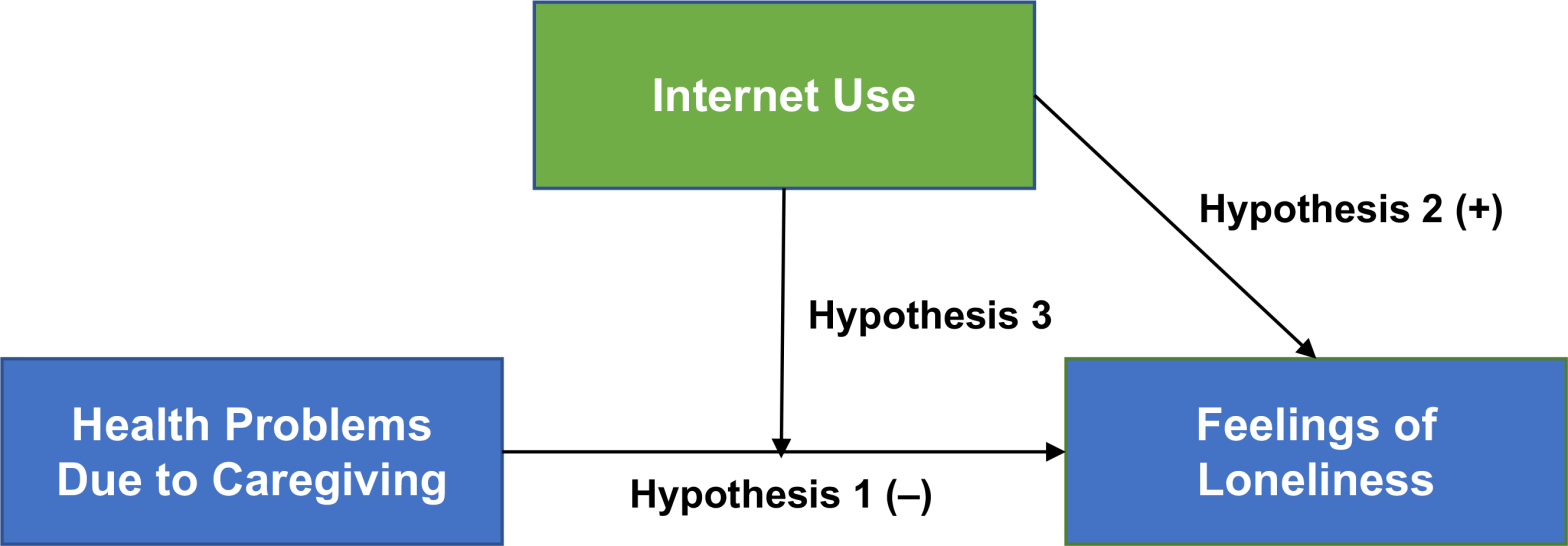
Conceptual framework.

This study tests three hypotheses: (1) experiencing adverse health effects from caregiving is associated with a higher level of loneliness; (2) frequent internet use is associated with a lower level of loneliness; and (3) internet use protects against the adverse caregiving-related health effects on feelings of loneliness among older informal caregivers. The conceptual framework with three hypotheses is shown in Figure 1.

## Methods

### Data Source and Sample Selection

This study used cross-sectional data from the 2019‐2020 California Health Interview Survey (CHIS), which is the largest statewide health survey in the United States [20]. CHIS uses a complex, multistage sampling design to collect comprehensive health-related information from California’s community-dwelling population [20,21]. The survey is conducted either online or by telephone and collects data on sociodemographic characteristics, health-related behaviors, health status, health care access, and utilization patterns. The 2019‐2020 CHIS surveyed 44,109 adults with a screened response rate of 72.0%, producing a sample that is representative of California’s diverse population. Notably, CHIS is conducted in multiple languages and uses sampling strategies to reach various racial and ethnic and socioeconomic groups, ensuring broad representativeness of the state’s population [20].

From the full CHIS sample, we restricted our analytic sample to respondents who met the following criteria: (1) aged 65 years or older; (2) reported providing unpaid help to family members or friends with illnesses or disabilities in the past year; and (3) had complete information on the key study variables (internet use, health effects of caregiving, and loneliness). After applying these criteria, our final analytic sample consisted of 3957 older informal caregivers.

### Measurement

#### Dependent Variable: Loneliness

Loneliness was assessed using the modified version of the validated 3-item UCLA Loneliness Scale [22], demonstrating good reliability and validity in older adult populations [4,23]. Participants responded to three questions: “How much of the time do you feel (1) lack of companionship, (2) left out, and (3) isolated from others?” Each item was rated on a 3-point scale: “1=hardly ever or never,” “2=some of the time,” and “3=often.” [22] Responses were summed to create a continuous loneliness score ranging from 3 to 9, with higher scores indicating greater levels of loneliness. The internal consistency of the 3-item UCLA Loneliness Scale in our sample was excellent (Cronbach α=0.974).

#### Independent Variables

*Health effects of caregiving*: The health impact of caregiving was measured by asking respondents “whether you had experienced physical or mental health problems as a result of their caregiving responsibilities in the past 12 months.” Responses were dichotomized (yes/no), with “yes” indicating the presence of caregiving-related health problems.

*Frequency of internet use*: Internet use was measured by asking participants the question: “how often you used the internet?” Responses were coded on a 4-point scale: “1=less than a few times a day,” “2=few times a day,” “3=many times a day,” and “4=almost constantly.” Following previous research [24], we treated internet use frequency as a continuous variable, with higher scores indicating more frequent use of the internet.

#### Covariates

We included several potential covariates based on established associations with caregiving, internet, and mental health among older adults [25-28]. Sociodemographic covariates included age (continuous, in years), sex (0=male; 1=female), race/ethnicity (1=non-Hispanic White; 2=non-Hispanic Black; 3=Hispanic or Latino; 4=non-Hispanic Asian; and 5=others), U.S. born (0=not born in the U.S.; 1=born in the US), education level (0=less than high school; 1=high school or above), economic status measured by family income relative to the federal poverty level (0=<200%; 1=≥200%), and marital status (0=married/living with partner; 1=widowed/separated/divorced/never married). We also controlled for health status, including self-rated health (measured on a 5-point Likert scale from 1=poor to 5=excellent) and the number of self-reported chronic conditions (ie, hypertension, asthma, diabetes, heart disease, vision/hearing impairment). Additionally, we included caregiving context variables that might influence the relationship between our primary variables of interest: experiencing financial stress due to caregiving (measured on a 4-point Likert scale from 1=extremely stressful to 4=not at all stressful), caregiving hours per week (ranged 1 to 125), the care recipient’s living arrangement (1=living alone; 2=living with caregiver or other family members; and 3=living in a nursing home/assisted-living facility/other living situations), relationship to the care recipient (1=spouse/partner; 2=parent/parent-in-law; 3=other relatives; and 4=nonrelative), and whether the caregiver supported the care recipient with Alzheimer disease and related dementia (0=nondementia caregiver; 1=dementia caregiver).

#### Statistical Analysis

We first conducted descriptive analyses of all study variables, presenting continuous variables as means (SD) and categorical variables as frequencies and percentages (%). To visually examine the relationship between internet use frequency and loneliness, we created violin plots displaying the distribution of UCLA Loneliness Scale scores across the four internet use frequency categories. We also performed one-way ANOVA tests with post hoc Tukey Honestly Significant Difference (HSD) comparisons to assess differences in mean loneliness scores across internet use frequency groups.

To test our hypotheses, we used a series of multivariable linear regression models with loneliness as the dependent variable. After adjustment for all covariates, Model 1 examined the association between caregiving-related health problems and loneliness. Model 2 examined the association between internet use and loneliness. Model 3 examined the associations of both caregiving-related health problems and internet use with loneliness. Model 4 incorporated an interaction term between caregiving-related health problems and internet use to assess whether internet use moderated the association between caregiving-related health problems and loneliness, while controlling for the same set of covariates.

To facilitate the interpretation of significant interaction effects, we conducted post hoc analyses and created visual representations showing the relationship between caregiving-related health problems and loneliness at different levels of internet use. We also calculated adjusted means of loneliness scores for different combinations of caregiving-related health problems and internet use frequency to illustrate the interaction effect.

All analyses incorporated the complex survey design features of CHIS, including stratification, clustering, and sampling weights, to ensure that results were representative of older informal caregivers in California. We addressed missing data using multiple imputations for covariates with missing values less than 5%; cases with missing values on key study variables were excluded as per our sample selection criteria. We conducted all statistical analyses using Stata MP (version 17.0; StataCorp, with a significance level set at *P*<.05 for all tests. All analyses were conducted by a multidisciplinary team with expertise in gerontology, nursing, and biostatistics, providing confidence in the study’s methodological rigor.

### Ethical Considerations

This study was exempt from the Institutional Review Board review at New York University as CHIS is a publicly available dataset.

## Results

### Participants’ Characteristics

Table 1 displays the sociodemographic characteristics, health status, and caregiving context of the 3957 older informal caregivers in California who participated in the study. The participants had a mean age of 72.46 years (SD 5.88), with 58.6% (n=2,319) being female. The sample was predominantly non-Hispanic White (78.6%, n=3,109), with 11.7% (n=462) born outside the United States. Regarding educational attainment, most participants (86.7%, n=3,432) had at least some college education, and the majority (83.0%, n=3,283) reported family income at or above 200% of the federal poverty level.

**Table 1.** Characteristics of participants from the California Health Interview Survey 2019‐2020.

Variables	Participants, (N=3957), n (%)
Sociodemographic variables	
Age (years old), mean (SD, range)	72.46 (5.88; 65-85)
Sex (female), n (%)	2319 (58.6)
Race/ethnicity, n (%)	
Non-Hispanic White	3109 (78.6)
Non-Hispanic Black	130 (3.3)
Hispanic/Latinx	357 (9.0)
Non-Hispanic Asian	263 (6.6)
Other	98 (2.5)
Born outside of the United States, n (%)	462 (11.7)
Education, n (%)	
<High school	525 (13.3)
≥ High school	3432 (86.7)
% Federal poverty level, n (%)	
<200%	674 (17.0)
≥200%	3283 (83.0)
Marital status, n (%)	
Married/living with partner	1780 (45.0)
Widowed/separated/divorced/never married	2177 (55.0)
Health status	
Smoking, n (%)	
Currently smoker	198 (5.0)
Quit smoking	1495 (37.8)
Never smoking	2264 (57.2)
Body mass index, mean (SD, range)	26.8 (6.11; 12.65‐69.35)
Self-rated health, mean (SD, range)	2.44 (0.96; 1-5)
Number of chronic conditions^a^, mean (SD, range)	1.19 (1.00; 0-5)
Caregiving context, n (%)	
Experience financial stress due to caregiving, continuous	3.47 (0.81; 1-4)
Caregiving hours per week	15.39 (21.24; 1-125)
Care recipient’s living condition, n (%)	
Living alone	1010 (25.5)
Living with caregivers or other family members	2069 (52.3)
Nursing home/assisted living facility/other situations	878 (22.2)
Relationship to the care recipient, n (%)	
Spouse/partner	1099 (27.8)
Parent/parent-in-law	971 (24.5)
Other relatives	925 (23.4)
Nonrelative	962 (24.3)
Dementia caregiver, n (%)	
Yes	911 (23.0)
No	3046 (77.0)
Internet use frequency, mean (SD; range)	2.71 (0.84; 1-4)
Have health problems due to caregiving, n (%)	475 (12.0)
Loneliness, mean (SD; range)	3.98 (1.41; 3‐9)

a Chronic conditions: hypertension, asthma, diabetes, heart disease, vision/hearing impairment.

In terms of caregiving context, participants reported low to moderate levels of financial stress due to caregiving, with a mean score of 3.47 (SD 0.81) on a 4-point scale, where higher scores indicate less stress. The mean caregiving hours per week were 15.39 (SD 21.24) on a scale ranging from 1 to 125. Approximately half (52.3%, n=2069) of the care recipients lived with the caregivers or other family members, while 1010 (25.5%) lived alone and 878 (22.2%) resided in nursing homes, assisted living facilities, or other living arrangements. The sample exhibited a fairly even distribution across caregiver relationship types: 27.8% (n=1099) were caring for spouses or partners, 24.5% (n=971) for parents or parents-in-law, 23.4% (n=925) for other relatives, and 24.3% (n=962) for nonrelatives such as friends or neighbors. In addition, a total of 911 (23.0%) care recipients had Alzheimer disease and related dementia. Notably, 12.0% (n=475) of the caregivers reported experiencing physical or mental health problems due to their caregiving responsibilities.

Regarding technology use, participants reported moderate levels of internet use, with a mean score of 2.71 (SD 0.84) on a 4-point scale, where a higher score indicates more frequent use. The mean UCLA Loneliness Scale score was 3.98 (SD 1.41) on a scale ranging from 3 to 9, indicating relatively low levels of loneliness in the overall sample.

### Associations Between Health Effects of Caregiving, Internet Use, and Loneliness

**Figure 2.**
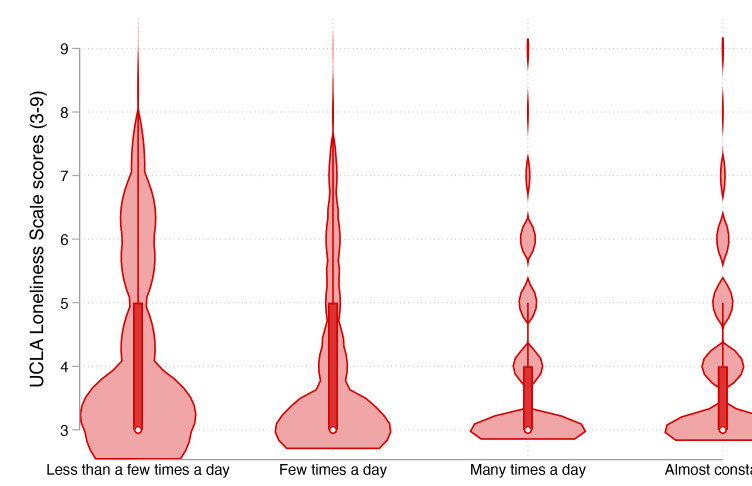
Violin plots of loneliness scores by internet use frequency categories among older informal caregivers (n=3957). The width of each violin represents the probability density of data at different values. White dots indicate medians, red boxes show interquartile ranges, and red lines represent the range excluding outliers. ANOVA testing confirmed significant differences between groups (*P*<.001). Mean and sample sizes: less than a few times a day (mean 4.14, SD1.49; n=188), few times a day (mean, SD 3.99, 1.47; n=1568), many times a day (mean 3.85, SD 1.33; n=1398), and almost constantly (mean 3.84, SD 1.27; n=803).

Figure 2 presents the violin plot depicting the distribution of loneliness scores across the four internet use frequency categories. The distribution shape changes notably across categories, with the ’less than a few times a day’ group showing the highest mean score of loneliness (4.14, SD 1.49) and the widest distribution, indicating greater variability in loneliness experience among infrequent internet users. In contrast, the ’almost constantly’ group exhibited the lowest median score (3.85, SD 1.27), with a narrower distribution concentrated toward lower loneliness values. One-way ANOVA confirmed significant differences in loneliness across the four categories (*F*_3, 3953_=7.05, *P*<.001). Post hoc tests revealed significant differences between all pairs of internet use frequency groups (all *Ps*<.05) except between the “many times a day” and “almost constantly” groups.

### Regression Analysis of Caregiving-Related Health Effects, Internet Use, and Their Interaction

In Model 1 (Table 2), after adjusting for sociodemographic characteristics, health status, and caregiving context variables, caregivers who reported experiencing physical or mental health problems due to caregiving had significantly higher loneliness scores compared to those who did not report such problems (*β*=0.76, SE 0.07, *P*<.001). The regression analysis in Model 2 also revealed a significant inverse relationship between internet use frequency and loneliness (*β*=−0.11, SE 0.03; *P*<.001). Specifically, more frequent Internet use was associated with a lower level of loneliness after controlling for socio-demographic factors, health status, and caregiving context variables (Model 3). Additionally, we conducted a sensitivity analysis excluding self-rated health and number of chronic conditions from the regression models; the association between caregiving-related health problems and loneliness remained significant and of similar magnitude, suggesting that our findings were not driven by adjusting for overall health status.

**Table 2.** Multivariable linear regression of internet use and health problems of caregiving with loneliness.

Variables	Model 1	Model 2	Model 3	Model 4
	β coefficient (SE)			
Health problems of caregiving	0.76 (0.07)^a^	--	0.75 (0.07)^a^	1.19 (0.21)^a^
Internet use frequency^b^	--	−0.11 (0.03)^a^	−0.10 (0.02)^a^	−0.08 (0.02)^a^
Health problems of caregiving × internet use	--	--	--	−0.16 (0.07)^c^
Age, continuous	−0.01(0.00)^c^	−0.01(0.00)^c^	−0.01(0.00)^c^	−0.01(0.00)^c^
Female (ref. Male)	0.07 (0.04)	0.12 (0.05)^d^	0.08 (0.04)	0.08 (0.04)
Race/ethnicity (ref. Non-Hispanic White)				
Non-Hispanic Black	−0.32 (0.09)^a^	−0.34 (0.09)^a^	−0.30 (0.09)^a^	−0.30 (0.09)^a^
Hispanic/Latino	−0.10 (0.06)	−0.08 (0.06)	−0.08 (0.06)	−0.09 (0.06)
Non-Hispanic Asian	−0.33 (0.09)^a^	−0.34 (0.09)^a^	−0.36 (0.09)^a^	−0.36 (0.09)^a^
Other	−0.16 (0.11)	−0.19 (0.11)	−0.14 (0.11)	−0.15 (0.11)
Born outside of the United States (ref. Born in the United States)	0.39 (0.07)^a^	0.36 (0.07)^a^	0.39 (0.07)^a^	0.39 (0.07)^a^
Education (ref.<High school)				
≥ High school	0.04 (0.05)	0.07 (0.05)	0.02 (0.05)	0.02 (0.05)
**%** Federal poverty level (ref.<200**%**)				
≥200%	−0.04 (0.05)	−0.03 (0.05)	−0.05 (0.05)	−0.05 (0.05)
Marital status (ref. Married/living with partner)				
Widowed/separated/divorced/never married	−0.57 (0.05)^a^	−0.60 (0.05)^a^	-0.56 (0.05)^a^	−0.57 (0.05)^a^
Smoking (ref. Currently smokes)				
Quit smoking	0.07 (0.10)	0.06 (0.10)	0.05 (0.10)	0.05 (0.10)
Never smoking	−0.06 (0.10)	−0.08 (0.10)	−0.09 (0.10)	−0.09 (0.10)
Body mass index, continuous	−0.01 (0.00)^c^	−0.01 (0.00)^d^	−0.01 (0.00)^c^	−0.01 (0.00)^c^
Self-rated health, continuous^e^	−0.16 (0.03)^a^	−0.21 (0.03)^a^	−0.17 (0.03)^a^	−0.17 (0.03)^a^
Number of chronic conditions^f^, continuous	0.02 (0.02)	0.01 (0.02)	0.02 (0.02)	0.02 (0.02)
Caregiving hours per week, continuous	−0.01 (0.00)^a^	−0.01 (0.00)^a^	−0.01 (0.00)^a^	−0.01 (0.00)^a^
Experience financial stress due to caregiving, continuous	0.23 (0.03)^a^	0.30 (0.03)^a^	0.23 (0.03)^a^	0.23 (0.03)^a^
Care recipient’s living condition (ref. Living alone)				
Living with caregivers or other family members	0.12 (0.06)^c^	0.11 (0.06)	0.11 (0.06)	0.11 (0.06)
Nursing home/assisted living facility/other situations	0.08 (0.07)	0.08 (0.07)	0.07 (0.07)	0.07 (0.07)
Relationship to the care recipient (ref. Spouse/partner)				
Parent/parent-in-law	−0.02 (0.07)	−0.05 (0.07)	−0.03 (0.07)	−0.02 (0.07)
Other relatives	0.00 (0.06)	−0.05 (0.06)	−0.01 (0.06)	−0.00 (0.06)
Dementia caregiver (ref. No)				
Yes	0.67 (0.15)^a^	0.59 (0.17)^a^	0.69 (0.16)^a^	0.72 (0.17)^a^

a *P*<.001.

b A higher score indicates more frequent Internet use.

c *P*<.05.

d *P*<.01.

e A higher score indicates better self-rated health (5-point Linkert Scale).

f Chronic conditions: hypertension, asthma, diabetes, heart disease, vision/hearing impairment.

To examine the moderating role of internet use on the association between caregiving-related health problems and loneliness, we included an interaction term between internet use and caregiving-related health problems in the multivariable regression model (Model 4 in Table 2). The interaction term was statistically significant (*β*=−.16, SE 0.07, *P*=.02), indicating that internet use significantly attenuated the negative impact of caregiving-related health problems on loneliness.

**Figure 3.**
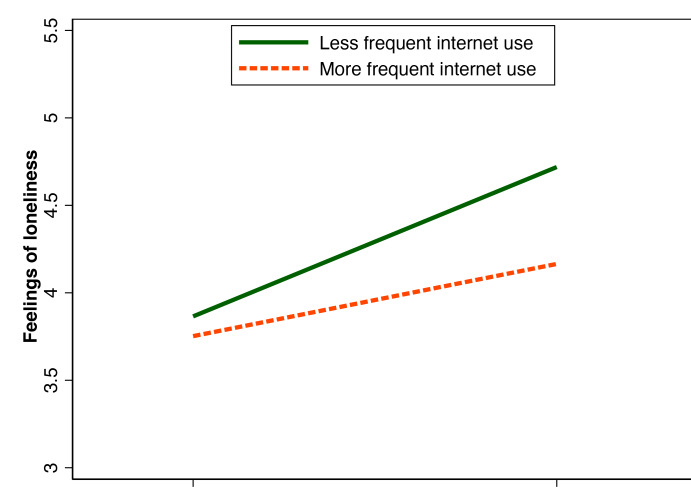
The moderating effect of internet use on the association between caregiving-related health problems and loneliness (California Health Interview Survey 2019‐2020). The model was controlled for sociodemographics, health status, and caregiving context characteristics.

Figure 3 illustrates this interaction effect, showing that the positive association between caregiving-related health problems and loneliness was stronger among caregivers with low internet use compared to those with high internet use. For caregivers with low internet use, experiencing health problems due to caregiving was associated with a substantial increase in loneliness scores. In contrast, for caregivers with high internet use, the difference in loneliness scores between those with and without caregiving-related health problems was less pronounced.

## Discussion

### Principal Findings

We hypothesized that older caregivers who experienced caregiving-related health problems would report higher loneliness, that more frequent internet use would be associated with lower loneliness, and that internet use would buffer the impact of caregiving-related health problems on loneliness. Consistent with these hypotheses, our study revealed three key findings regarding loneliness among older informal caregivers. First, caregivers who reported experiencing physical or mental health problems due to caregiving had significantly higher levels of loneliness compared to those who did not report such problems, supporting our first hypothesis. Second, more frequent internet use was associated with lower levels of loneliness, confirming our second hypothesis. Third, internet use significantly moderated the relationship between caregiving-related health problems and loneliness, supporting our third hypothesis.

The observed association between caregiving-related health problems and increased loneliness aligns with previous research on the mental health consequences of caregiving burden [29-31]. Loneliness among older caregivers can stem from various sources, including emotional loneliness within the relationship with their care recipients, especially if the care recipient has dementia [9]. The nature of caregiving often necessitates significant time commitment and can lead to social isolation, as caregivers frequently sacrifice their social lives and personal needs to provide care [7,31]. This isolation can be particularly pronounced for spousal caregivers, who typically bear the most intensive care duties and often experience diminished ability to participate in social activities. The continuous concern for their care recipients can result in a sacrifice of personal freedom and a decline in their sense of identity within societal roles [30].

Our finding that more frequent internet use is associated with lower loneliness scores among older caregivers contributes to the growing literature on the potential benefits of digital technology for this population [15,25]. This relationship may reflect the ability of internet-based communication to facilitate social connections despite the time and mobility constraints that often accompany caregiving responsibilities [32]. The internet can serve as a bridge that enhances the intergenerational connections, mitigates feelings of loneliness among older informal caregivers, and boosts their overall quality of life [15,25]. By mitigating space and time constraints, the internet provides a channel through which caregivers can boost interaction within their interpersonal networks, alleviate perceived isolation, and maintain good mental status [33]. These benefits may be particularly valuable for older caregivers who face challenges in maintaining in-person social connections due to their caregiving duties. Research has shown that technology-enabled support interventions can increase feelings of social bond, social support, and shared experiences [25,33,34], all of which can alleviate loneliness and its negative effects on mental health among older adults. Digital platforms can also foster social and coping skills and help change maladaptive perceptions and thoughts, thereby decreasing loneliness (eg, evidence from internet-based caregiver support interventions shows improved coping and reduced isolation) [33,34]. Our findings regarding the association between internet use and a lower level of loneliness among older caregivers align with these mechanisms.

Additionally, our study found that internet use moderates the association between caregiving-related health problems and loneliness. This suggests that internet use may serve as a protective factor against loneliness for caregivers experiencing health problems due to their caregiving responsibilities. Consistent with the Stress Process Model’s framework—which suggests that coping resources buffer the negative effects of caregiving stressors on outcomes like loneliness – our findings indicate that frequent internet use likely served as a coping resource that attenuated the impact of caregiving-related health problems on loneliness [19]. The internet may offer dual benefits for older caregivers experiencing health problems, as it can serve both as a source of health information and a means of maintaining social connections despite physical limitations or time constraints [25,26,33,34]. Digital technology can enable caregivers to access health resources, connect with health care providers, and join support groups relevant to their specific health concerns, while simultaneously facilitating social interaction with friends and family.

Our findings have several implications for interventions targeting loneliness among older caregivers. First, they suggest that promoting internet use among older caregivers may be a promising strategy for reducing loneliness, particularly for those experiencing health problems due to caregiving. Improving digital health literacy is crucial for enabling older caregivers to effectively utilize online resources. Recent evidence shows that tailored interventions can significantly enhance eHealth literacy in older populations, thereby empowering caregivers to find, evaluate, and apply online health information [35,36]. Health care providers should consider assessing both caregiving-related health problems and internet use patterns when evaluating the risk of loneliness among older caregivers. Those with caregiving-related health problems and low internet use may be particularly vulnerable to loneliness and could benefit from targeted interventions. Moreover, policy makers should consider investing in infrastructure and programs that increase internet accessibility and digital literacy among older adults, particularly those with caregiving responsibilities. This could include subsidized internet access, device distribution programs, and community-based digital literacy training tailored to older adults. Government, policy makers, and society should prioritize their efforts to promote internet literacy among older informal caregivers, advance the development of internet and digital infrastructure, and bridge the urban-rural divides.

This study has several limitations that need to be acknowledged. First, CHIS’s cross-sectional design precludes causal inferences regarding the relationships between caregiving-related health problems, internet use, and loneliness. Longitudinal studies are needed to examine how changes in caregiving-related health problems and internet use over time influence trajectories of loneliness among older caregivers. Second, our measure of internet use focused solely on frequency rather than specific types of internet activities or duration of online interactions. Future research should examine how different patterns and durations of internet use (eg, social media, entertainment, health information seeking) may differentially affect loneliness among older caregivers. Understanding internet use patterns and device adoption across older caregivers and care recipients is increasingly recognized in enhancing geriatric care [37]. Third, health problems due to caregiving measures are self-reported and may not exclusively reflect problems caused by caregiving alone. It is challenging to definitively attribute health issues solely to caregiving responsibilities with a cross-sectional self-report item, and we caution that this variable should be interpreted as a perceived impact of caregiving on health. Finally, although our sample was representative of older informal caregivers in California, the generalizability of our findings to other geographic contexts may be limited. Future studies should examine these relationships in diverse populations and settings, including rural areas with limited internet infrastructure and communities with varying levels of technological integration. Future research should explore the development of tailored digital interventions specifically designed to address loneliness among older caregivers. These interventions could integrate components addressing both caregiving-related stressors and social connection, potentially leveraging artificial intelligence to personalize content based on individual caregiving contexts and preferences. In addition, emerging technologies such as social robots have shown promise in supporting older people with dementia and their informal caregivers [38] and could be explored as complementary tools to reduce loneliness in caregiving contexts.

### Conclusion

Our study provides evidence that internet use is associated with lower levels of loneliness among older informal caregivers and could buffer against the negative impact of caregiving-related health problems on loneliness. This study highlights the potential of digital technology as a tool for addressing loneliness in this vulnerable population. As the population ages and the number of older caregivers increases, developing effective strategies to support their psychosocial well-being becomes increasingly important. Internet-based interventions represent a promising approach, particularly for caregivers facing health challenges related to caregiving responsibilities. Future research and policy efforts should focus on enhancing internet accessibility, promoting digital literacy, and developing age-friendly digital platforms that meet the specific needs of older caregivers.

## Abbreviations

CHIS: California Health Interview Survey
HSD: Honestly Significant Difference
